# Arctic circulation regimes

**DOI:** 10.1098/rsta.2014.0160

**Published:** 2015-10-13

**Authors:** Andrey Proshutinsky, Dmitry Dukhovskoy, Mary-Louise Timmermans, Richard Krishfield, Jonathan L. Bamber

**Affiliations:** 1 Physical Oceanography Department, Woods Hole Oceanographic Institution, Woods Hole, MA, USA; 2 Center for Ocean-Atmospheric Prediction Studies, Florida State University, Tallahassee, FL, USA; 3 Department of Geology and Geophysics, Yale University, New Haven, CT, USA; 4 School of Geographical Sciences, University of Bristol, Bristol, UK

**Keywords:** arctic climate variability, circulation regimes, freshwater and heat content

## Abstract

Between 1948 and 1996, mean annual environmental parameters in the Arctic experienced a well-pronounced decadal variability with two basic circulation patterns: cyclonic and anticyclonic alternating at 5 to 7 year intervals. During cyclonic regimes, low sea-level atmospheric pressure (SLP) dominated over the Arctic Ocean driving sea ice and the upper ocean counterclockwise; the Arctic atmosphere was relatively warm and humid, and freshwater flux from the Arctic Ocean towards the subarctic seas was intensified. By contrast, during anticylonic circulation regimes, high SLP dominated driving sea ice and the upper ocean clockwise. Meanwhile, the atmosphere was cold and dry and the freshwater flux from the Arctic to the subarctic seas was reduced. Since 1997, however, the Arctic system has been under the influence of an anticyclonic circulation regime (17 years) with a set of environmental parameters that are atypical for this regime. We discuss a hypothesis explaining the causes and mechanisms regulating the intensity and duration of Arctic circulation regimes, and speculate how changes in freshwater fluxes from the Arctic Ocean and Greenland impact environmental conditions and interrupt their decadal variability.

## 1. Introduction

In this paper, we build on previous investigations of Arctic Ocean wind-driven circulation regimes [1–6] and their transformations under rapidly changing Arctic conditions, and extend them by focusing on identifying the causes and mechanisms responsible for decadal variability—in particular, on the deviation from climatological decadal variability since 1997. Atmospheric circulation, ice drift and wind-driven ocean motions are dynamically connected and responsible for significant changes in the state and variability of Arctic environmental parameters. The presence of relatively mobile sea ice, driven by winds and ocean currents, and regulating atmosphere/ocean interactions is a key factor influencing Arctic climate and changes on seasonal, interannual and decadal time scales. This has been recognized since Nansen's era [7] of Arctic exploration, and studied and discussed in numerous publications (e.g. [8–11]). In the following, we review past studies of Arctic decadal variability and circulation-regime definitions to introduce our hypothesis which may explain the breakdown of previously observed regular decadal variability.

### (a) Arctic climate changes: 1948–2013

A representative suite of various Arctic climate data are available from approximately 1948; these observations have been summarized recently (e.g. [12,13]) to show that during the last two to three decades Arctic atmosphere and ocean temperatures have increased, sea ice volume and extent have decreased, permafrost has thawed, storminess has increased, sea level has risen and biological processes have become more complex and diverse [13].

**Figure 1.**
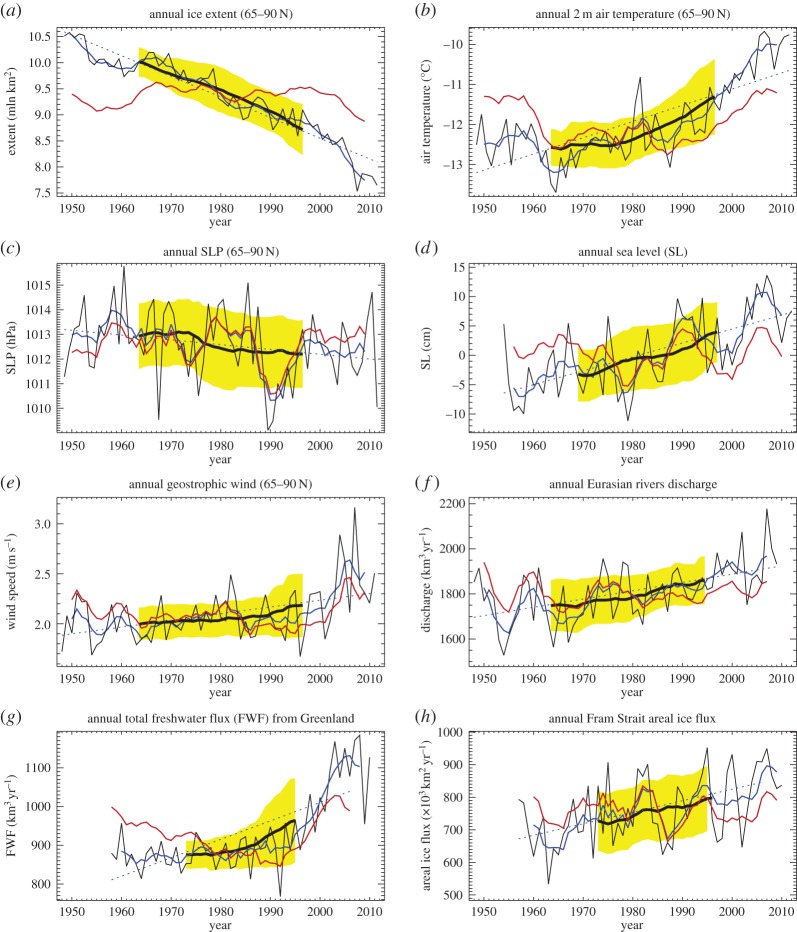
(*a*) Timeseries of annual ice extent, (*b*) 2 m air temperature, (*c*) SLP, (*d*) sea level, (*e*) geostrophic wind, (*f*) Eurasian river runoff, (*g*) freshwater flux from Greenland and (*h*) Fram Strait areal ice flux (sources of data are shown in table 1). Thin black line depicts annual, blue line shows 5-year running mean, red line is the detrended 5-year running mean, thick black line shows climatic changes as the 30-year running mean, dotted line is the 1948–2013 linear trend, and the yellow shaded area shows±1 s.d. of the 30-year mean series.

We begin with an assessment of the 1948–2013 time series of Arctic environmental parameters, including sea ice extent, air temperature, sea-level atmospheric pressure (SLP), sea level, geostrophic wind, river discharge, Greenland freshwater flux and areal ice flux via Fram Strait (figure 1; for data sources, see table 1). We apply a 30-year running mean to all of the annual data (black lines in figure 1) to examine general trends in these parameters with the understanding that ‘climate’ is defined as weather averaged over a 30-year period (thick black lines in figure 1) following World Meteorological Organization and IPCC [17] recommendations. This averaging provides information on climate changes from approximately 1963 to 1998, and demonstrates that the Arctic climatological annual values of: (a) sea ice extent reduced by about 14%, (b) air temperature increased by 10% (from −12.55°C in 1963 to −11.30°C in 1998), (c) SLP reduced by approximately 0.7 hPa; (d) sea level rose by approximately 0.06 m; (e) geostrophic wind speed over the Arctic Ocean increased by about 15%; (f) Eurasian river runoff increased by 14%; (g) freshwater flux from Greenland has increased rapidly since the 1980s with acceleration after 1997 (present values are around 60% of the magnitude of Eurasian river discharge); and (h) areal ice flux via Fram Strait increased by 15% which must have had a significant influence on the total ice volume in the Arctic Ocean and specifically on the minimum of ice extent in September [18].

**Table 1.** Data sources.

data	data description and data source
SLP	four times daily derived from NCAR/NCEP reanalysis product [14]. *Reanalysis data provided by the NOAA/OAR/ESRL PSD, Boulder, CO, USA, from their website at* http://www.esrl.noaa.gov/psd/
sea ice extent	monthly 25 km grid resolution product from NSIDC derived by Comiso 1999, updated 2012. Bootstrap sea ice concentrations from Nimbus-7 SMMR and DMSP SSM/I-SSMIS (26 October 1978 through 31 December 2011). Boulder, CO, USA: National Snow and Ice Data Center. Digital media
2 m air temperature	monthly values for 1948/01–present. *NCEP reanalysis*[14]. *Derived data provided by the NOAA/OAR/ESRL PSD, Boulder, CO, USA, from their website at* http://www.esrl.noaa.gov/psd/
Eurasian rivers discharge	monthly 1935–2011 discharges from Ob', Yenisey, Lena, Severnaya Dvina, Pechora and Kolyma. The data were provided by A. I. Shiklomanov (Water Systems Analysis Group Institute for the Study of Earth, Oceans, and Space, University of New Hampshire, Durham, NH 03824). Data are also available online from the US Geological Survey (http://waterdata.usgs.gov/ak/nwis) and Environment Canada (http://www.wsc.ec.gc.ca/applications/H2O/index-eng.cfm)
sea level	monthly relative sea-level data. Approximately 70 tide-gauge stations in the Barents and Siberian Seas (Kara, Laptev, East Siberian and Chukchi Seas) have recorded sea-level changes from the 1950s through the 2000s.These data are available for model validation at the Permanent Service for Mean Sea-Level archive (http://www.pol.ac.uk/psmsl/pub/nucat.dat) and at the Woods Hole Oceanographic Institution website (http://www.whoi.edu/science/PO/arcticsealevel)
Greenland freshwater flux	monthly freshwater fluxes from Greenland [15]
areal ice flux via Fram Strait	1957–2010 monthly ice area flux from [16] and timeseries received directly from L. H. Smedsrud (2011, personal communication)

**Figure 2.**
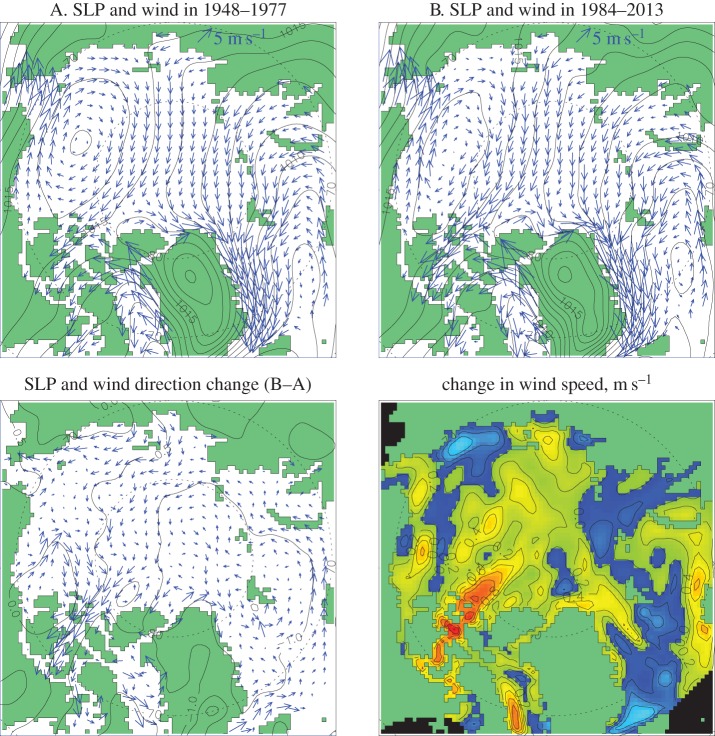
Upper panels show climatic annual SLP (hPa) and geostrophic wind (m s^−1^) over 1948–1977 (A) and over 1984–2013 (B). Bottom panels show differences in SLP and geostrophic wind direction between B and A climates (left) and change in geostrophic wind speed (right). Reduction of wind speed is depicted in bluish colours.

Changes in the atmospheric circulation between the climate state of 1948–1977 and that of 1984–2013 (centred, with 30-year mean averaging, on the years 1963 and 1998, respectively) are not visually dramatic (figure 2, upper panels) although there is a tendency to intensification of cyclonic atmospheric motion due to a reduction of SLP over the Arctic Ocean, and a reduction in area of the Arctic High centred over the Beaufort Gyre region (figure 2, left bottom panel). Mean geostrophic wind is intensified along the Norwegian coast (figure 2, right bottom panel) apparently increasing the Atlantic water inflow (and heat transport) to the Barents Sea [19]. At the same time, winds are stronger in the latter period in the Fram Strait region, increasing the sea-ice flux from the Arctic Ocean towards the Greenland Sea (e.g. [20]) and in the northern Canadian Archipelago straits, potentially increasing the ice and water flux from the Arctic Ocean towards Baffin Bay and the Labrador Sea [21].

The preceding analysis corroborates the general agreement among scientists that the Arctic has been experiencing changes in the major environmental parameters, and many studies have addressed the observed changes, climate feedbacks and their consequences (e.g. [18,22,23]). However, there remains an incomplete understanding of the mechanisms responsible for the observed changes on decadal time scales. Figure 1 highlights significant variability of all parameters on a range of time scales shorter than 30 years, occurring in the Arctic since 1948. Trends and variability in most parameters appear to have accelerated relatively recently (since approx. 1980s–1990s; figure 1), and already influence climate curves shown in figure 1 (compare observed and detrended timeseries). However, we cannot estimate the present (2014) climate state until we have observations spanning 2000 to 2029. In this sense for example, the fate of Arctic sea ice for at least the next decade remains highly indeterminant despite predictions of a seasonally ice-free Arctic in the near future [24–26]. This problem of uncertainty is well known and is due to short (on climatic time scales) observational records and longer natural variability of the Arctic climate system.

### (b) Decadal change indices and motivation

Consistent with figure 1, many studies (e.g. [27–30]) indicate Arctic climate variability on a wide range of time scales, with seasonal, decadal and multi-decadal peaks (table 2). This apparent presence of variability at statistically significant frequencies (table 3) suggests potential for predictability of Arctic conditions. There has long been motivation to find basic mechanisms that cause the observed changes in the Arctic atmosphere–ice–ocean system (e.g. [31–38]) at decadal and shorter time scales. Climate indices such as the North Atlantic Oscillation (NAO; e.g. [39,40]), Arctic Ocean Oscillation (AOO) [1–4] and Arctic Oscillation (AO) [41] have been constructed to characterize complex climate processes and explain past variability. However, attempts to predict the NAO and AO cycles were not successful (e.g. [42–44]). This study investigates causes and mechanisms regulating the intensity and duration of Arctic circulation regimes and puts forward an explanation for the well-pronounced decadal changes in the region during the 1948–1996 period and the apparent cessation of the quasi-decadal cycle of circulation regimes after 1996.

**Table 2.** Results of spectral and wavelet analysis, where IE is sea ice extent; T, 2 m air temperature; SLP, sea-level pressure; SL, sea level; R, Eurasian river discharge; F, Fram Strait ice area flux; G, total Greenland freshwater flux; AOO, AOO index. Crosses (×) mean that peak is significant with greater than 50% and less than 90% confidence interval and bold crosses (**×**) depict peaks with confidence interval greater than 90%.

period (years)	IE	T	SLP	SL	R	F	G	AOO
22–19	×	**×**	**×**	**×**			**×**	
16–14		**×**						**×**
13–11		**×**			**×**	**×**		
10–7	×		×	**×**		×		**×**
6–3		×	×	×	×	**×**		×
2–0.75	**×**	×			×	×	**×**

**Table 3.** Linear correlation coefficients among 5-year annual running mean climate parameters timeseries for 1960–2009, where IE is sea ice extent; T, 2 m air temperature; SLP, annual mean SLP over the Arctic Ocean; SL, sea level; R, Eurasian river discharge; G, total Greenland freshwater flux; F, Fram Strait ice area flux; AO, Arctic Oscillation index; AOO, AOO index. Bold font depicts correlation coefficients for detrended timeseries shown in figure 1.

	IE	T	SLP	SL	R	G	F	AO	AOO
IE	1.00	−0.94	0.21	−0.82	−0.86	−0.83	−0.81	−0.53	0.42
	**1.00**	−**0.60**	−**0.21**	−**0.30**	−**0.31**	−**0.40**	−**0.28**	**0.07**	−**0.55**
T	−0.94	1.00	−0.05	0.75	0.86	0.92	0.82	0.38	0.53
	−**0.60**	**1.00**	**0.37**	**0.09**	**0.44**	**0.66**	**0.36**	−**0.25**	**0.61**
SLP	−0.82	−0.05	1.00	−0.52	−0.15	0.07	−0.23	−0.88	0.63
	−**0.21**	**0.37**	**1.00**	−**0.52**	**0.15**	**0.49**	−**0.02**	−**0.92**	**0.76**
SL	−0.82	0.75	−0.52	1.00	0.60	0.75	0.67	0.67	0.20
	−**0.30**	**0.09**	−**0.52**	**1.00**	−**0.23**	**0.33**	**0.13**	**0.42**	−**0.07**
R	−0.86	0.86	−0.15	0.60	1.00	0.76	0.66	0.51	0.42
	−**0.31**	**0.44**	**0.15**	−**0.23**	**1.00**	**0.26**	**0.00**	**0.10**	**0.34**
G	−0.83	0.92	0.07	0.75	0.76	1.00	0.63	0.23	0.69
	−**0.40**	**0.66**	**0.49**	**0.33**	**0.26**	**1.00**	**0.03**	−**0.44**	**0.76**
F	−0.81	0.82	−0.23	0.67	0.67	0.63	1.00	0.47	0.21
	−**0.28**	**0.35**	−**0.02**	**0.13**	**0.00**	**0.03**	**1.00**	**0.05**	−**0.05**
AO	−0.53	0.38	−0.88	0.67	0.51	0.23	0.47	1.00	−0.38
	**0.07**	−**0.25**	−**0.92**	**0.42**	**0.10**	−**0.44**	**0.05**	**1.00**	−**0.67**
AOO	0.42	0.53	0.63	0.20	0.42	0.69	0.21	−0.38	1.00
	−**0.55**	**0.61**	**0.76**	−**0.07**	**0.34**	**0.76**	−**0.05**	−**0.67**	**1.00**

Compared to the NAO and AO, the AOO index, defined on the basis of a wind-driven simulated sea surface height field across the Arctic [1], is a more Arctic-centric index. The index is a measure of the intensity and sense (clockwise/anticyclonic or counterclockwise/cyclonic) of the Arctic Ocean wind-driven upper oceanic circulation. Here, we use this index to identify and explain mechanisms regulating changes in Arctic circulation regimes and environmental parameters at decadal time scales. Over 1948–1996, the AOO has been shown to be the most appropriate index to capture variability in key Arctic environmental parameters [2,45,46]. After 1996, many previously established correlations between climate indices and environmental parameters no longer appear to apply. For example, after 1996, despite a low positive or neutral AO index, a strong anticyclonic circulation regime (ACCR) has persisted over the Arctic Ocean instead of the more cyclonic circulation as would be expected from previous correlations between the AO index and sea-ice drift direction [47,48]. At the same time, the Arctic experienced continued warming and loss of sea ice extent and volume [49].

In §2, we review the AOO index, updating definitions and results of Proshutinsky *et al*. [1–3] and Dukhovskoy *et al*. [4], explain its physical meaning, and demonstrate that this index accurately reflects changes in the Arctic's wind-driven circulation and well reproduces existing observational data. In §3, the major anomalies of ACCR and cyclonic circulation regime (CCR) are analysed and decadal variability of the circulation regimes is investigated. We discuss the unusually long ACCR dominating Arctic conditions since 1997 until present in §4 and employ box-model experiments in §5 to speculate on causes of this deviation from expected behaviour. In §6, we summarize and conclude.

## 2. Arctic Ocean Oscillation index

The AOO was first defined in [1] based on analysis of annual sea surface height fields simulated employing a regional Arctic shallow-water barotropic-coupled ice–ocean model [1,50]. The model was forced by 6-hourly wind stresses derived from NCAR/NCEP atmospheric reanalysis data (table 2) for 1948–1993 and was developed initially to investigate and predict storm surges and tides in the Arctic Ocean [50]. It has been employed since 1992 by the Arctic and Antarctic Research Institute (St Petersburg, Russia) as an operational model to predict sea ice conditions and sea-level variability in the Arctic Ocean. Although only a shallow-water model, full testing and calibration against observed sea-level timeseries along the Siberian coastline and sea-ice drift data from the International Arctic Buoy programme prove that the model well represents the principal patterns of sea ice dynamics, sea-level variability at coastal tide gauges and ocean circulation [51–53].

**Figure 3.**
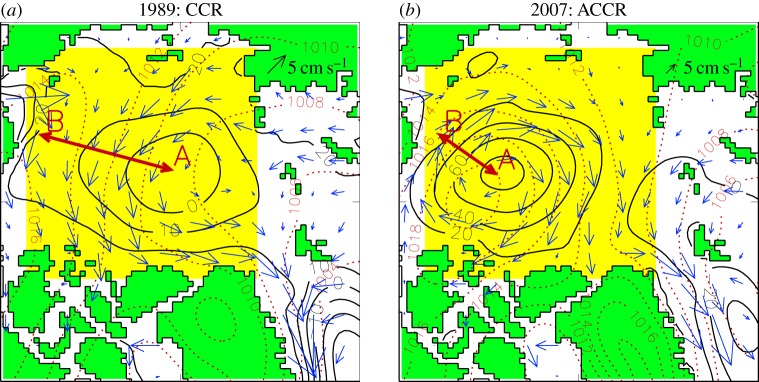
Illustration of the AOO index definition and physical meaning. Annual simulated sea surface heights (cm, contour interval is 10 cm) and ice drift (blue arrows) under CCR (*a*) and ACCR (*b*) wind forcing. Red dotted isolines depict SLP (hPa, contour interval is 2 hPa). Yellow box depicts region occupied by Arctic High—major atmospheric circulation feature over the Arctic Ocean. The red arrows in the yellow box show how gradients of sea surface heights (AOO indices) are calculated: differences between sea surface heights in the centre (A) and periphery of the closed circulation (B) are divided by the distance between chosen sea surface height isolines. Note that AOO index is non-dimensional. Anticyclonic circulation has positive sea surface height gradients (AOO indices), while cyclonic circulation has negative gradients (AOO indices).

**Figure 4.**
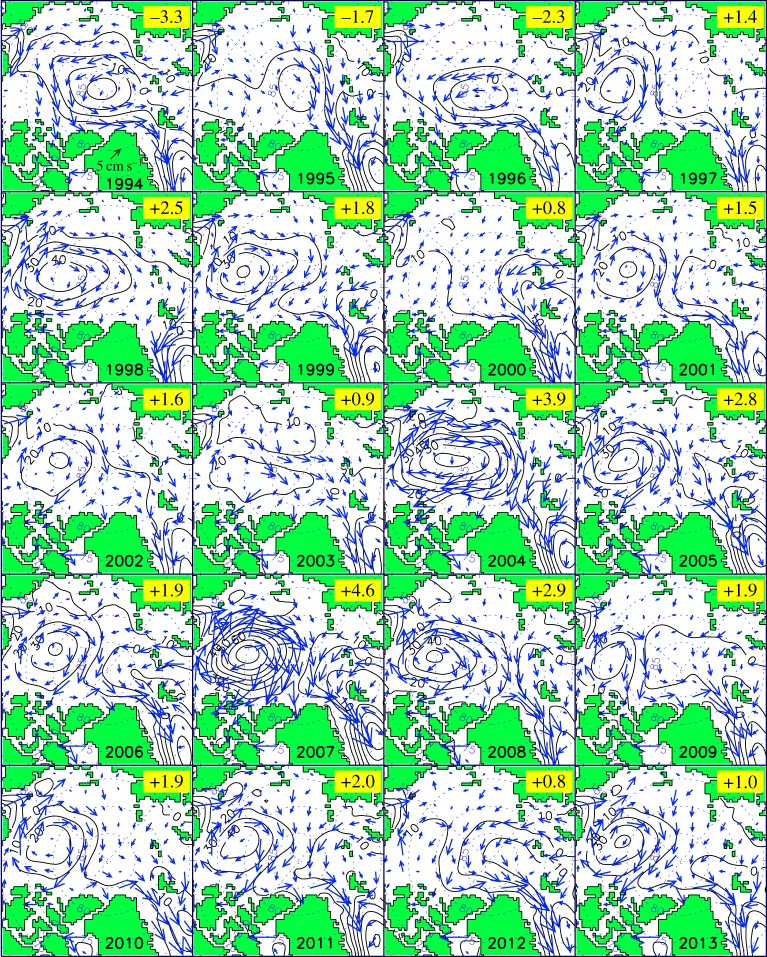
Simulated annual sea surface heights (black contour lines, cm) and wind-forced ice drift component (blue arrows) after 1993. The sea surface height contour interval is 5 cm. The value of the AOO index is shown in the top right corner of all panels. Annual sea surface heights and wind-forced ice drift patterns for 1946–1993 are shown in [1].

To calculate the AOO index [1], the location of the extremum in the annual simulated sea level in the Arctic Ocean region is first located and then the last closed sea surface height isoline around that extremum is found to determine the sea surface height horizontal gradient (this number (×10^−6^) is non-dimensional and its calculation procedure is illustrated in figure 3). While the magnitude of the gradient is somewhat sensitive to the shape of the last closed isoline, the sign of the gradient is robust. Positive values of these gradients correspond to a raised central sea surface and anticyclonic (clockwise) water circulation (figure 3*b*). Negative values correspond to lowered central sea surface and cyclonic (counterclockwise) water circulation (figure 3*a*). Fig. 8 in [1] illustrates the mean annual simulated sea surface heights in the Arctic Ocean for 1948–1993. Figure 4 in this paper shows annual circulation patterns and AOO indices for 1994–2013.

**Figure 5.**
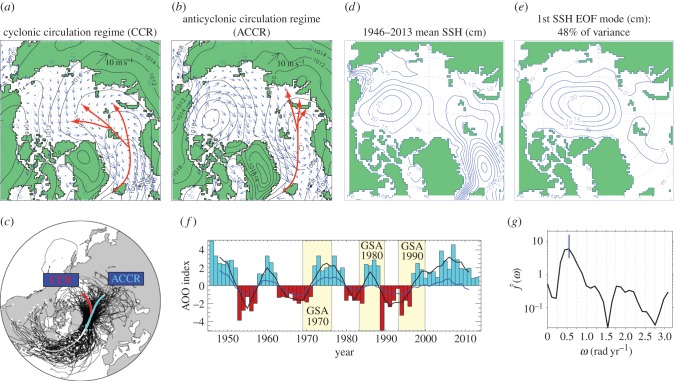
AOO index and circulation regimes. Panels (*a*) and (*b*) show typical annual distributions of SLP and surface winds for cyclonic and anticyclonic atmospheric circulation regimes, respectively; red arrows show prevailing cyclone tracks, summarized from panel (*c*). (*c*) Greenland Sea cyclone trajectories for 1949–2002 (fig. 2 from [54]). White dots indicate the start of each cyclone trajectory and the white and grey lines show two different paths identified by cluster analysis and well supporting SLP distributions corresponding to circulation regimes shown in (*a*) and (*b*), respectively. This means that annual SLP distribution patterns express statistics of cyclone counts—prevailing cyclone trajectories. (*d*) 1946–2013 mean wind-forced simulated sea surface height (SSH) distribution. (*e*) First EOF mode (48% of the variance) of the annual sea surface height pattern. (*f*) AOO indices. The thick black line depicts the 5-year-running-mean timeseries of the AOO index derived manually following the approach of Proshutinsky & Johnson [1]. Positive (blue bars) AOO indices correspond to years with ACCRs and negative (red) bars show cyclonic regimes. Coefficients of the annual first EOF mode of the simulated wind-driven sea surface heights as the 5-year running mean are shown as the blue solid line. GSA years are shown as shaded boxes. (*g*) Spectra of the AOO calculated from the timeseries of the detrended annual AOO index from 1946 to 2013 using the Tukey window with band width 0.279 rad yr^−1^. The vertical blue bar denotes the 95% confidence interval of the peak with corresponding frequency *ω*=0.551 rad yr^−1^ (11.4 years).

An objective measure of the AOO index timeseries employs empirical orthogonal function analysis (EOF) to derive the major modes of variability for annual sea surface height values for 1948–2013. The first EOF mode of the sea surface height pattern (figure 5*e*,*f*) describes 48% of the annual sea surface height variability for this period. Differences in amplitude between the EOF-based AOO and that derived following the manual approach [1,2] are to be expected, because the EOF-based index describes only 48% of variability, while the manually derived AOO measures the full strength of the cyclonic/anticyclonic circulation. Note that these differences change none of the general results here, and the two methods yield indices that are highly correlated (*r*=0.89) and demonstrate the dominant decadal signal of change in the Arctic Ocean wind-driven circulation regime. The manually derived AOO is analysed here.

## 3. Arctic Ocean Oscillation variability, circulation regimes and their characteristics

The AOO strongly correlates with many environmental parameters [1,2], characterizing interannual change in the Canada Basin [3], Beaufort Gyre freshwater content [55,56] and variability of freshwater content across the entire Arctic Ocean [57]. During negative AOO years (red bars in figure 5*f*), the Arctic atmosphere is relatively warm and humid due to frequent penetration of cyclones from the North Atlantic (figure 5*a*,*c*) into the central Arctic [1,2]. This is consistent with results of [54] (figure 5*c*), where it was shown that cyclones that originate in the North Atlantic are a major source of moisture and heat to the Arctic. These cyclones have two distinct trajectories bringing moisture into the central Arctic or Siberia, depending on the atmospheric circulation regime. In the regime discussed above (hereinafter, CCR), low SLP dominates over the central Arctic Ocean (figure 5*a*), with winds forcing the sea ice to drift cyclonically (counterclockwise). During a CCR, both exports of sea ice (the solid component of the freshwater flux) and low salinity polar water (liquid freshwater flux) to the Greenland–Iceland–Norwegian (GIN) Sea increase [1,2]. Anomalously high freshwater flux results in freshening of the upper GIN Sea. The Great Salinity Anomalies (GSAs) observed in the North Atlantic Subpolar Region (NASR) in the 1970s, 1980s and 1990s (figure 5*f*; e.g. [58–60]) correlate well with the history of the advent of CCRs starting several years after the initiation of CCRs over the Arctic Ocean.

By contrast, during positive AOO years (figure 5*f*, blue bars), the trajectories of North Atlantic cyclones are shifted eastward resulting in fewer cyclones reaching the central Arctic (figure 5*b*,*c*). In this climate regime (hereinafter, ACCR), high SLP dominates over the Arctic with anticyclonic winds forcing sea ice to drift clockwise (figure 5*b*). During a typical ACCR, the Arctic atmosphere is relatively cool and dry [1,2]. Lower-than-normal air temperatures and anticyclonic winds lead to thicker ice and increased ice extent in an ACCR compared with a CCR. During an ACCR, freshwater is accumulated in the Beaufort Gyre of the Canada Basin [3,55] due to Ekman transport convergence, reducing freshwater transport towards the NASR (which includes the Labrador Sea and Nordic Seas). This could lead to reduced stratification of the upper ocean there, promoting deep convection and atmospheric warming [3,4].

Results of Proshutinsky *et al*. [2] showed that the two-climate regime theory characterized by AOO variability explains much of the observed decadal variability of the Arctic Ocean and helps to reconcile the different conclusions between analyses of environment data obtained during different climate regimes. Recently, the AOO index was used in several studies to explain decadal-scale regime shifts in northwest Atlantic shelf ecosystems [61] and understand climatic regulations of growth rates of bivalve shells around Svalbard and in the northwestern Barents Sea (e.g. [62]). Our studies also show that there are significant differences in seasonal variability between years with CCRs and ACCRs [45].

Timeseries of the AOO index from 1948 to 1996 indicate that ACCRs and CCRs alternate at approximately 5- to 7-year intervals with a period of quasi-oscillation of about 10–15 years. In a stark deviation from this pattern, the ACCR started in 1997 has dominated the Arctic over the last 17 years (figure 5*f*). Of essential importance to our understanding of the evolving Arctic system, and its impact on the global environment, is to discern the causes and consequences of the apparent breakdown in the decadal variability of circulation regimes. Why has the well-pronounced decadal variability observed in 1948–1996 been replaced by relatively weak interannual changes under ACCR conditions after 1996?

## 4. Conceptual models of Arctic climate variability

Several conceptual models of Arctic climate decadal variability have been introduced since the 1990s [63–66] and recently in [3–6]. Mechanisms for multi-decadal Arctic change have also been studied [29,67], but here we focus in particular on decadal variability. One key element of decadal-change conceptual models is the freshwater flux from the Arctic Ocean to the North Atlantic, and another is the atmospheric heat flux from the North Atlantic to the Arctic. Compelling manifestations of the link between the Arctic and the North Atlantic are: (i) salinity anomalies that originated in the Arctic [58,59,68] and propagated in the subpolar gyre in the 1970s, 1980s and 1990s as GSAs [58,59] and (ii) atmospheric warming and cooling events that are related to the intensity of cyclone activity in the central and eastern Arctic [69,70] and coupled to the Arctic atmospheric circulation [1,41].

To explain the observed decadal changes in Arctic climate, a conceptual hypothesis [3] was formulated. In this hypothesis, the freshwater and heat exchanges between the Arctic Ocean and North Atlantic are self-regulated and their interactions are realized via decadal auto-oscillations. Based on this work, an idealized multi-box model of the Arctic Ocean and NASR ocean–ice–atmosphere system was developed and employed [4–6] to demonstrate how the system oscillates between an ACCR and a CCR.

A detailed description of the multi-box model is provided in [5], while only a brief outline follows here. The prognostic model consists of two coupled modules: an Arctic Ocean and a NASR module, where each includes a coupled sea ice–ocean box model and an atmospheric model. The sea ice–ocean model consists of a thermodynamic ice model and a mixed layer-pycnocline model, with prognostic variables: water temperature and salinity, mixed layer depth and sea-ice thickness. Ocean heat and salt fluxes are specified at the open boundaries of the two modules (Bering Strait, rivers and North Atlantic), and fluxes between the modules are calculated from simulated temperature, salinity and the density gradient between the Arctic and NASR. The atmosphere in the Arctic model is treated via an energy balance, and surface air temperature in the NASR module is calculated as modelled temperature anomaly superimposed on a climatological daily value. The atmospheric heat flux between NASR and Arctic modules is taken to be proportional to the temperature difference between the two atmospheric modules. The full model is forced by daily solar radiation, wind stress, and river runoff. For validation purposes, results from model experiments reproducing seasonal and decadal variability of the major system parameters were analysed and compared with observations and other models [5].

It is obvious that such a multi-box model has several limitations: it does not include dynamics of the atmosphere, ice and ocean; the modelled system is closed, and there are no influences from the global ocean and atmosphere (this is addressed below in §5); and the model cannot show pathways of freshwater from the Arctic Ocean to the subpolar convective gyres. Nevertheless, the advantage of the box model is its simple formulation that allows one to investigate the basic relationships between components of the studied climate system. This task is not straightforward with coupled atmosphere–ice–ocean models, where extricating the role of internal mechanisms in climate shifts is complicated by too many other factors.

**Figure 6.**
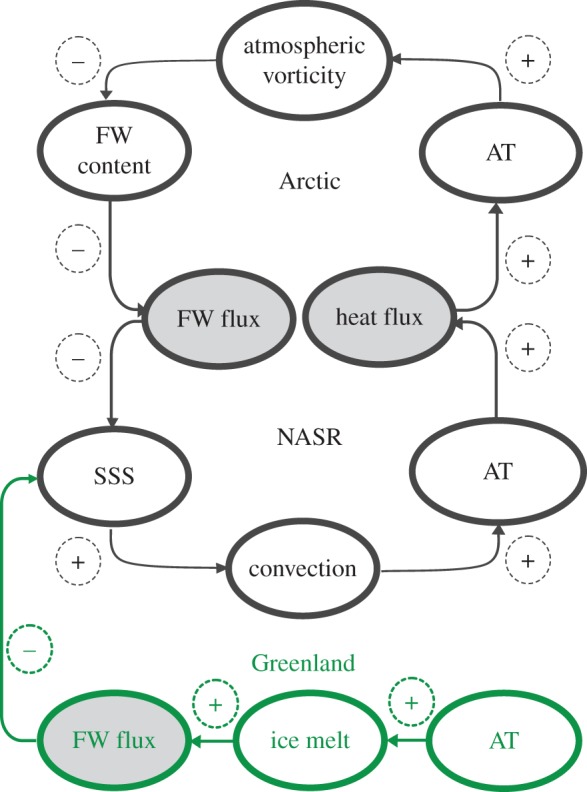
Arctic–NASR idealized atmosphere–ice–ocean climate system with feedbacks regulating decadal variability. A plus sign denotes mechanisms with positive feedback between two cells (i.e. an increase/decrease in one cell causes an increase/decrease in the second), a minus sign denotes mechanisms with negative feedback (i.e. an increase/decrease in one cell causes a decrease/increase in the second). Portions in black represent the idealized behaviour of a closed system under stable climate conditions. Green portions of the schematic represent the influence of FW fluxes from Greenland on parameters of the Arctic–NASR system. For this case, the new system is not closed. AT, SSS and FW stand for air temperature, sea surface salinity and freshwater, respectively.

It is important to note that in this multi-box model, regime shifts are controlled by atmospheric heat fluxes from the NASR and freshwater fluxes from the Arctic Ocean. The idealized Arctic–NASR climate system (figure 6) correctly shows that the reduced atmospheric heat advection to the Arctic results in lower-than-normal Arctic atmospheric temperature, higher-than-normal SLP and negative atmospheric vorticity (ACCR). ACCR wind forcing leads to freshwater accumulation in the Arctic Ocean via processes of Ekman convergence and reduction of freshwater flux to the NASR, which increases sea surface salinity in the NASR and promotes intensification of deep convection preconditioned by weaker water column stability [3,71]. During this regime, the NASR releases heat accumulated in the deep ocean layers to the atmosphere leading to a warmer-than-normal atmosphere and to the intensification of cyclogenesis. This atmospheric heat is transported to the Arctic with cyclones and contributes to a positive atmospheric vorticity (CCR) over the Arctic Ocean due to the substantial reduction in the Arctic SLP. This state lasts approximately 5–7 years (half period of oscillation). During the second half of this oscillation (5–7 years), positive atmospheric vorticity (cyclonic winds) forces increased freshwater flux from the Arctic Ocean to the NASR and reduces NASR surface salinity. Consequently, strengthened upper ocean stratification leads to reduced NASR convection, reduced heat flux from the ocean to the atmosphere and from the NASR towards the Arctic Ocean, and the Arctic circulation regime shifts to an ACCR. The ACCR is characterized by predominantly negative vorticity and colder than normal Arctic and NASR atmospheres.

Solutions obtained in the Arctic Ocean and NASR box model [4–6] well reproduced the observed evolution of the major anomalies in the ocean temperature and salinity structure, sea ice volume and freshwater fluxes during ACCRs and CCRs prior to approximately 1997. However, after 1997, the real climate system (figures 1 and 5*f*) does not behave according to the predictions of the conceptual models described above [3–6]. After 1997, the AOO index is positive (figures 4 and 5*f*) and exhibits only weak interannual variability associated with the strength of the ACCR with no shift in annual mean conditions from an ACCR to CCR. Similarly, other conceptual models cited above do not have a mechanism to explain and predict the recent large-scale anticyclonic circulation observed in the Arctic.

## 5. Increased Greenland freshwater flux as a mechanism for regime shift cessation

Following our conceptual model, the prevailing ACCR should have switched to a CCR in the early 2000s. Instead, the Arctic has been characterized by an ACCR since 1997 at least until 2013 (figure 5*f*). Results of the Beaufort Gyre Observing System [55,56,72] indicate anomalously high freshwater accumulation (5000 km^3^ relative to reference salinity of 34.8) in the Beaufort Gyre since the start of the present ACCR in 1997. At the same time, the GIN Sea (as part of the NASR) shows a warming of the deep layers in the 2000s that has been attributed to a cessation of deep convection in the region [73]—conditions that typically develop during a CCR when freshwater is released from the Arctic Ocean. Following our conceptual model, one would expect enhanced deep convection and cyclone formation over the NASR (enabled by reduced freshwater fluxes from the Arctic) would have resulted in a regime shift from ACCR to CCR nearly a decade ago due to freshwater release from the Arctic Ocean. However, analyses of freshwater fluxes through Fram Strait [74,75] purport that the Fram Strait annual mean freshwater flux does not show any large variations since 2002.

We hypothesize that there is at least one additional factor, neglected in the multi-box model formalism, which has been influencing NASR conditions and has disrupted the auto-oscillatory decadal AOO index variability. The discussed Arctic Ocean–NASR system was viewed as a closed system (the black portions in figure 6) but in recent decades, anomalously warm atmospheric temperatures have led to increased Greenland melt, driving an important external forcing to the Arctic Ocean–NASR system (figure 1*g* and green portions in figure 6). Recent assessments of freshwater flux from Greenland show that during 1992–2010, this flux to the Arctic Ocean and NASR increased by 36% [15]. We speculate that the excess freshwater advected into the NASR may have significant impact to deep convection (with subsequent atmospheric cooling and reduction of cyclonic activity). The effect would be to impede the decadal oscillations that were a feature of the observations and well represented by our idealized multi-box model (without a contribution to freshwater from Greenland) prior to the 2000s. Figure 6 (green portions) represents the influence of freshwater fluxes from Greenland on other environmental parameters in the Arctic–NASR system via reduction of NASR surface salinity, suppressing deep convection and maintaining a negative atmospheric vorticity (ACCR) over the Arctic.

To examine this central idea, we ran a multi-box model simulation incorporating the additional Greenland freshwater source. After 50 years, an additional freshwater flux is superimposed (following an appropriate seasonal cycle) on the total NASR freshwater input; the annual average of the additional freshwater flux is comparable to the estimated annual inflow from Greenland melt (approx. 46 km^3^ yr^−1^ [15]). We then ran a second simulation doubling the freshwater flux anomaly to the NASR box.

**Figure 7.**
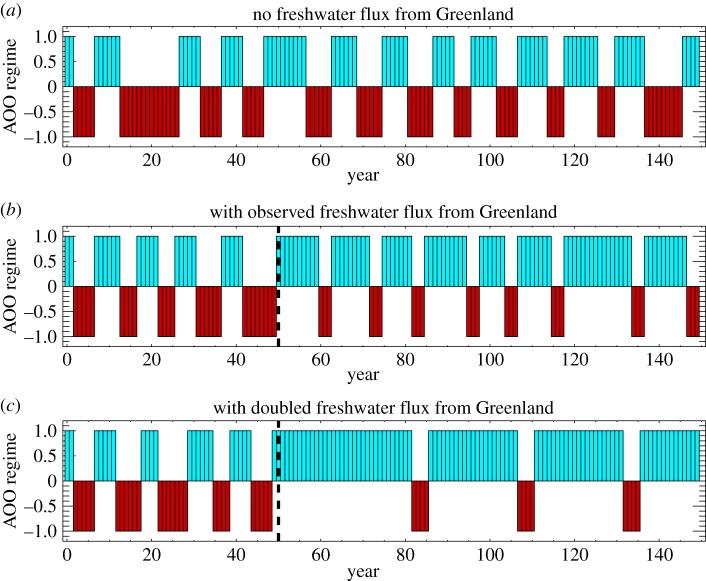
Duration of circulation regimes as simulated by the box model. Blue and red bars indicate ACCR and CCR years, respectively. Panels show model runs without (*a*), with observed (*b*) and doubled (*c*) freshwater flux from Greenland. The black vertical dashed line marks the start of the Greenland freshwater flux addition.

The control run with no freshwater flux from Greenland produces quasi-decadal oscillations between ACCRs and CCRs (figure 7*a*) that are similar to AOO variability (figure 5*f*) until 2002, while the additional freshwater flux resulted in significantly longer ACCRs (figure 7*b*), consistent with observations after 1996. A doubling of the volume flux of freshwater (figure 7*c*) from Greenland to the NASR (not inconceivable in the future given present warming and Greenland melting trends [76,77]) resulted in extreme changes in the system behaviour with ACCRs dominating for more than three decades, separated by CCRs with 3–4 year duration. These idealized modelling results are consistent with our broad hypothesis that additional freshwater fluxes from Greenland melt may be sufficient to suppress previously established Arctic decadal variability.

In the experiments described above, freshening of the upper NASR plays the key role in disrupting the auto-oscillatory behaviour of the Arctic Ocean–NASR system. An additional freshwater flux from Greenland may impact water column stability and suppress convection in the NASR, reducing air–sea heat fluxes and leading to lower than normal atmospheric temperature in the NASR and Arctic Ocean. Freshening of the upper NASR also acts to maintain the small dynamic height difference between the Arctic Ocean and the NASR (and to reduce the potential for freshwater release from the Arctic Ocean to the NASR) and within the generally accepted view of convection processes in the NASR (e.g. [68,78–80]).

There have been several numerical studies, employing models having a range of complexities and resolutions, to investigate the role of freshwater flux increase from Greenland in the intensity of the Atlantic meridional overturning circulation [81–83]. Results show that depending on model resolution, applied forcing and duration of simulation, the influences of Greenland ice melt on the Atlantic meridional overturning circulation and on the Arctic and NASR conditions differ substantially. In general, however, results of these model studies indicate that freshwater release from Greenland weakens or interrupts decadal variability but mechanisms and processes responsible for decadal changes are not discussed.

## 6. Future scenario for circulation regimes

Based on the analysis presented here, we speculate that:

— ocean–atmosphere heat fluxes in the NASR vary with Arctic circulation regimes and regulate interactions between the Arctic Ocean and NASR. Ocean to atmosphere heat fluxes in the NASR are larger during ACCRs (compared with CCRs) supporting cyclogenesis and ultimately a regime shift to a CCR;
— the duration of ACCRs and CCRs in a changing climate will be different from those in the twentieth century; a new mode of variability in the Arctic may consist of long-duration ACCRs, separated by relatively short-duration CCRs; and
— the major cause of cessation of decadal variability is the monotonically increasing freshwater flux anomaly from Greenland that began in the mid-1990s.

One important uncertainty in our analysis is that available time series observations are not sufficiently long to verify the hypothesized causality of the Arctic climate variability and Greenland melt (which itself varies on multi-decadal time scales [76,77]). Nevertheless, the data together with idealized modelling and physical context provide compelling evidence that input of fresh Greenland melt to the surface high-latitude regions can interrupt decadal variability of the Arctic–NASR system.

While most predictions of future Arctic climate under global warming are done by extending observed trends in environmental variables, the effects of global warming are likely not to be monotonic due to complex feedbacks and relationships between Arctic environmental parameters, as shown here. We speculate that under the new scenario Greenland would continue melting and supporting longer duration ACCRs, which could result in Arctic cooling accompanied by increased ice extent and thickness—similar to conditions observed in the 1970s. Enhanced upper ocean stratification in the NASR could continue to inhibit heat exchange between the ocean and atmosphere and, subsequently, transport of heat by cyclones to the Arctic. This would have broad-reaching climate implications by limiting the advection of heat from the mid-latitudes and is therefore a negative feedback on Arctic amplification under global warming.

## Data accessibility

All datasets are publicly available and data sources are listed in table 1.
